# Exhaled Breath Temperature Home Monitoring to Detect NSCLC Relapse: Results from a Pilot Study

**DOI:** 10.1155/2022/1515274

**Published:** 2022-02-21

**Authors:** Giovanna Elisiana Carpagnano, Todor A. Popov, Giulia Scioscia, Nicoletta Pia Ardò, Donato Lacedonia, Mario Malerba, Pasquale Tondo, Piera Soccio, Domenico Loizzi, Maria Pia Foschino Barbaro, Francesco Sollitto

**Affiliations:** ^1^Department of Basic Medical Sciences, Neuroscience and Sense Organs, Section of Respiratory Disease, University “Aldo Moro” of Bari, Bari, Italy; ^2^University Hospital “Sv. Ivan Rilski”, Urvich St., 13, 1612 Sofia, Bulgaria; ^3^Department of Medical and Surgical Sciences, University of Foggia, Italy; ^4^Institute of Respiratory Disease, Policlinico Riuniti of Foggia, Foggia, Italy; ^5^Thoracic Surgery Section, Department of Medical and Surgical Sciences, University of Foggia, Italy; ^6^Department of Translational Medicine-Piemonte Orientale University, Italy

## Abstract

**Background:**

Exhaled breath temperature (EBT) has been shown to reflect airway inflammation as well as increased vascularization, both involved in the pathogenesis of lung cancer. The aim of this study was to look for evidence that continuous EBT monitoring by such a device may help the early detection of relapse of lung cancer in patients with NSCLC who have been subjected to surgery with radical intent. *Case Series*. We included 11 subjects, who had been subjected to lung resection with radical intent for NSCLC in a prospective observational study. All patients received individual devices for EBT measurement and used them daily for 24 months after surgery. Subjects were also followed up by means of regular standard-of-care clinical and radiologic monitoring for lung cancer at four intervals separated by 6 months (T0, T1, T2, T3, and T4). In 5 patients, relapse of lung cancer was documented by means of lung biopsies. All of them recorded an elevation of their EBT at least one-time interval (T1), corresponding to 6 months, before the relapse was diagnosed at T4. The individual EBT graphs over time differed among these patients, and their mean EBT variability increased by +4% towards the end of 24 months of monitoring. By contrast, patients without a relapse did not document an elevation of their EBT and their variability decreased by -1.4%.

**Conclusions:**

Our pilot study provided evidence that continuous EBT monitoring can help in the early detection of lung cancer relapse.

## 1. Introduction

Presently, lung cancer is still the leading cause of cancer death worldwide. Global numbers are continuously rising despite an ongoing small decline in the Western world [1].

Tumor-node-metastasis (TNM) clinical staging system is considered the milestone for predicting prognosis, and the surgical removal of the tumor (anatomical resection with systematic nodal dissection) is the standard treatment for operable patients [2].

Patients affected by non-small-cell lung cancer (NSCLC), treated with a surgical radical intent, have a significantly increased risk to develop a lung cancer relapse or a second primary lung tumor. Regarding the Italian population, the last report of the “Associazione Italiana di Oncologia Medica” (AIOM) declared that 1/3 of lung cancer patients who underwent radical intent surgery developed a relapse [3].

In a large group of patients who underwent surgical resection, the standardized follow-up revealed that during the first 4 years after surgery, the risk of recurrence ranges from 6% to 10% per person per year, but thereafter, it decreases to 2% [4]. Within this period, a relapse pattern can be recognized: during the first 2 years, a local relapse is prevalent; between the second and the fourth year, recurrence is dominated by distant metastases [5]; after the fifth year, the probability of having a relapse is virtually absent. The risk of developing a second primary lung cancer follows a more uniform pattern over time, ranging from 1 to 6% per person per year, and does not diminish all along [4, 6].

Boyd et al. examined the timing of local and distant relapses among 250 of 975 patients who underwent surgery for primary lung cancer, and 17%, 44%, and 39% of the recurrences were, respectively, local, distant, and combined [7].

Several sets of data support the existence of a gene expression profile predicting survival and risk of cancer relapse [8, 9]. Histological studies have demonstrated that invasive growth of perilesional and intratumoral blood vessels plays a pivotal role in cancer recurrence [10]. This feature is referred to as neovascularization and is an essential phase for tumor growth, both in case of recurrence or in *de novo* tumors.

Furthermore, it is known that cancer cells secrete mediators that stimulate neoangiogenesis and hence growth of the cancer. The increased vascularity of the airways as a consequence of neoangiogenesis alters the thermal balance in the lungs and contributes to the upregulation of airway temperature and tone [11, 12].

Using X-halo Pro® device (Delmedica Investments, Singapore) to noninvasively measure EBT, our group has previously demonstrated that the temperature increase in airways is a pathophysiologic result of lung cancer development [13, 14]. This precision device allowed the assessment of EBT in a noninvasive and user-friendly way. This “gentle” approach is particularly useful in thoracic oncology, where patients are usually stressed, depressed, and uncooperative. A new model developed specifically for home monitoring, X-halo Home® (Delmedica Investments, Singapore), shortens the time of EBT measurement and transfers the data of the measurements to a specialized site ensuring secure storage and remote counseling by the treating physician. Although X-halo Home® has been developed with the purpose of monitoring asthma, the rationale of using it to detect increased thermal energy production because of neoangiogenesis underlying recurrent tumor growth seems quite feasible.

We hypothesized that continuous home monitoring of patients having undergone prior radical intent surgery for NSCLC would detect a slope of increasing EBT if recurrent or *de novo* tumor growth occurs. Hence, the aim of our study was to check whether continuous daily measurement of EBT could early detect a relapse of lung cancer in radically operated patients.

## 2. Case Series

This study was conducted in accordance with the STROBE statement for prospective observational studies [15]. Written informed consent was obtained from all subjects, and approval was granted by the Institutional Ethics Committee of the University of Foggia.

We conducted a prospective, single-center, observational follow-up study of 11 consecutive patients who underwent surgical radical resection for a NSCLC, from June 2015 and January 2016, at the Unit of Thoracic Surgery of the University of Foggia (Italy) (*n* = 132/2013).

All patients were given a personal X-halo Home device (Delmedica Investments, Singapore) and were instructed how to measure their EBT at the comfort of their homes for a period of 24 months. The data from the device had been uploaded every week on a specialized site and subsequently analyzed.

All patients were referred for anatomic lung resection and systematic lymphadenectomy, according to the international guidelines [2, 16–18].

EBT assessments had been conducted in the morning before taking in food and medications. Patients visited the clinic for standard follow-up procedures, consisting of a physical examination at 3-6-12-18-24 months and contrast-enhanced chest and upper abdomen CT scans every 6 months during the first two years [2, 3, 16–18].

Data collected during the follow-up were used to allot patients to two groups: group R included those patients with a radiological and local/distant relapse or new primary lung tumors, and group non-R includes the patients without a relapse whatsoever.

**Figure 1:**
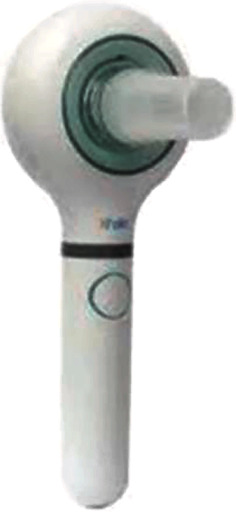
“X-halo Home” device.

EBT was measured with an X-halo Home (Delmedica Investments, Singapore) according to manufacturer's instructions (http://www.xhalocare.com) (Figure 1).

Briefly, subjects had to download the smart phone application called “X-halo Home” on their smart phones and to follow the instructions on the display of their smart phones, inhaling through the nose and exhaling into the device via a mouth piece; they had to perform 8 breathing cycles to see their actual EBT. The device records the discrete time points of the incremental temperature curve and transmits them to the X-halo Home website. The study investigators could log onto the website using personal credentials and identification number of the patient's device and retrieve the data needed for analysis (Figure 1).

The EBT values were recorded at the enrollment (T0, at the first outpatient check, 15 days after surgery) and then daily for the 24 months of follow-up. Intermediate time points of averaged EBT measurements were set at month 6 (T1), month 12 (T2), month 18 (T3), and month 24 (T4). The results were expressed as means ± standard deviation (SD) for normally distributed continuous variables, as median, first, and third quartile (Q1 and Q3) for continuous variables of skewed distribution, and as frequencies (percentage) for categorical variables. Differences in continuous variables were analyzed using an independent two-tailed *T*-test or the Mann–Whitney *U* test, as appropriate. Categorical variables were handled using the chi-square test or Fisher exact test when necessary. A two-tailed level of significance of *P* < 0.05 was accepted. Data were analyzed using GraphPad for MAC.

All initially enrolled 11 patients having undergone radical intent surgical treatment for NSCLC completed the two-year follow-up. The characteristics of patients are presented in Table 1.

The types of surgical procedures were lobectomy (*n* = 8), bilobectomy (*n* = 1), pneumonectomy (*n* = 1), and segmentectomy (*n* = 1). Histology revealed 7 adenocarcinomas and 4 squamous cell carcinomas. Pathological stages (according to the Eighth Edition of the IASLC TNM Staging System for Lung Cancer) were IA (*n* = 4), IB (*n* = 2), IIA (*n* = 1), and IIIA (*n* = 4). Three patients received adjuvant chemotherapy and one patient received radiation therapy.

**Figure 2:**
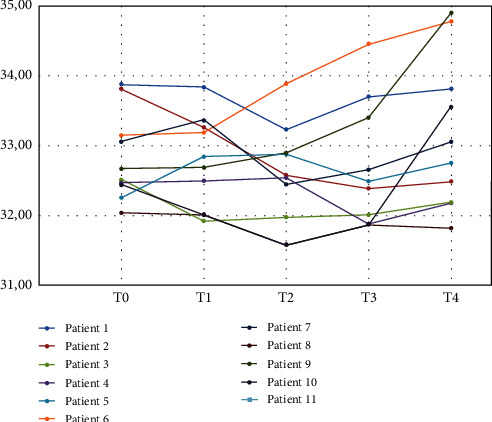
EBT °C at T0-T1-T2-T3-T4 in all patients enrolled.

When all patients were analyzed together, no differences in mean EBT ± SD at T0-T1-T2-T3-T4 (32.8 ± 0.6°C vs 32.7 ± 0.7°C vs 32.5 ± 0.7°C vs 32.8 ± 0.9°C vs 33.3 ± 1.1°C; NS) were uncovered (Table 2 and Figure 2).

Within 24 months of follow-up, based on the gold standard clinical, radiological, and histological (biopsies) criteria, 5 patients were diagnosed with tumor relapse (group R): local inpatients 9R, 10R, and 11R and at distant relapse of NSCLC inpatients 6R and7R (Table 3). In 4 of the group R patients, we documented a significant increase of EBT at least one month before the relapse was established, which then remained at this higher level during subsequent daily measurements. The individual patterns of the course of EBT in these patients were rather different. More specifically, patient 6R with distant relapse had at month 24 a mean increase of +1.63°C; patients 9R-10R-11R with local tumor relapse had at month 24 a mean increase of EBT of +2.37°C, +2.23°C, and +1.11°C, respectively; patient 7R had a somewhat higher EBT to begin with, which went down and surged again, +1.21°C before the distant relapse was diagnosed.

Patients without relapse (group non-R) showed a decrease of EBT during the follow-ups. In this group, only the patient 5 non-R evidenced a slight increase of EBT of +0.49°C.

Mean EBT variation during the follow-up was +1.4% for the whole group of patients: -1% for the group non-R and +4% for the group R.

## 3. Discussion

Inflammation is an inherent feature of transformation of cancer cells, with neoangiogenesis being essential for cancer evolution and diffusion [19]. Exhaled breath condensate and EBT have been proposed as noninvasive diagnostic tests for evaluation of inflammation in asthma and, only recently, in lung cancer [13]. An increase in EBT was recorded in patients with NSCLC compared to controls [14]. Our group has demonstrated a correlation between vascular endothelial growth factor (VEGF) and increase of EBT in patients with lung cancer, suggesting that airway neoangiogenesis drives an increase of EBT [13].

In our previous proof of concept cross-sectional studies, we have demonstrated that one-time measurement of EBT is significantly elevated in subjects with NSCLC. In the present study, we hypothesized that regular measurement of EBT during follow-up of patients having undergone radical surgical resection for NSCLC can detect local tumor relapse or de novo appearance early. This was made possible by the release of a newer technique for EBT measurement using individual devices, X-halo Home®, for home monitoring. These devices have been designed to record day-to-day fluctuations in EBT and airway inflammation, respectively, in subjects with asthma and chronic obstructive pulmonary disease (COPD) and to predict imminent episodes of exacerbation or harmful effects of occupational hazards, air pollution, or viral infections. The prospective design we implemented was unique in the sense that patients did daily measurements during two years. Contrary to our expectations, none of the recruited patients opted for discontinuation of the measurements.

However, the most noteworthy result of our study was the increase of EBT in the patients with tumor relapse within the 24 months following the surgical resection, which occurred before the relapse had been diagnosed with the standard-of-care methods by six months.

The observed relapse rate in our study was higher than expected considering prevalence and percentages recently published by AIOM in the Italian population [3]. This was probably due to the fact that we enrolled consecutive patients, and two of them had a positive oncological history with prior lung cancer resection (being a relapse or a second tumor), and four presented a locally advanced stage of tumor (IIIA).

There are tumor markers, such as CEA, that have been demonstrated to be independent factors predicting the risk of recurrence [20]. Nevertheless, the establishment of useful early markers is necessary to accurately identify a relapse.

An increase of body temperature as expression of mammarian cancer and melanoma due to disregulated cell growth and to hypervascularization has been previously described with termographic techniques based on noninvasive infrared imaging [21–23]. The location of lung tumors inside the chest, though, does not allow the thermographic approach. In our cohort, the surrogate marker of neoangiogenesis and inflammation was EBT measured over the span of 24 months. There were three interesting features of the results we obtained:

- Subjects who did not show a relapse of cancer had a smooth course of their EBT with a trend towards slight drop towards the end of the second year of monitoring, while the patients with lung cancer relapse recorded an increase of EBT at least one month before the radiologic and anatomopathological diagnosis of the relapse
- The EBT increase was more evident in those patients with a local recurrence and slighter in those with distant metastasis
- The variability of EBT in the nonrecurrence group was much lower than in the recurrence group (-1% for the group non-R vs. +4% for the group R)

This earlier increase in EBT than the manifestation of the radiological changes can be explained with the “silent” embryological processes that drive the development and growth of cancer which can be detected earlier than the critical tumor mass that can be detected radiologically. Our group also recently contributed to the validation of the EBT giving values in healthy subjects [24]. This facilitates the recognition of lung cancer also on the basis of single point EBT measurement, which we describe in our previous studies [13, 14]. However, the home monitoring of EBT with an individual device adds another dimension to our diagnostic capabilities, by introducing the possibility to make judgment based on time trends. This should be also viewed in the context of the common features between chronic obstructive lung diseases and lung cancer: monitoring the state of control of smokers with COPD could also detect a turning point in the EBT values suggestive of tumor growth.

Whatever the lung cancer treatment modality chosen, surgery or chemo- or radiation therapy, the initiation of earlier therapy with the early detection of recurrences could improve the outcome [25], compared with the therapy given when the patient becomes symptomatic from the tumor [2, 26–28]. This type of noninvasive, but intensive, follow-up may improve patient survival by detecting recurrences at an asymptomatic stage after surgery for NSCLC. Our next step would be to establish a more precise cut-off of EBT increase for a significant prediction of cancer relapse in a larger population. The currently available follow-up tools such as CT are only applicable at 6-month intervals due to radiation hazards. Alternative EBT daily measurement approach could capture the tumor growth at much more discrete time interval in patients resected for NSCLC.

If our results are confirmed and refined in larger studies, EBT monitoring could be incorporated as a follow-up tool in the scheme of follow-up, according to the international guidelines for radically treated NSCLC. If daily (or weekly) EBT measurements give rise to suspicion for tumor recurrence, CT could be performed ahead of schedule with ensuing initiation of earlier treatment of a recurrence, rendering better prognostic chances to patients with NSCLC relapse.

The small number of subjects in our study is a limitation of this study, which should be considered a proof-of-concept one. Larger trials would allow better correlations with the type of recurrence (local versus distant) and determination of sensitivity and specificity/receiver operating characteristic (ROC) curves. Another limit of this study is the duration of the follow-up, considering that the relapses are mainly observed at 4 years: a longer follow-up would allow to capture all relapse cases.

Furthermore, it remains to be seen whether different types of flare-ups, frequently seen in COPD patients, can trigger EBT changes, although with a probably different time course.

## 4. Conclusions

In conclusion, our results give us ground to believe that EBT daily monitoring could help the early identification of lung cancer relapse. Larger future studies could confirm and detail our preliminary findings. The potential of the noninvasive approach EBT monitoring to predict forthcoming relapse ahead of the development of clinical symptoms will certainly be to the benefit of the patients and would also have economic benefits for the healthcare system.

## Figures and Tables

**Table 1:** Anthropometric and clinical characteristics of patients.

Age (mean) (yr)	62.9 (range 29-77)
Gender (male/female)	9 : 2
Smoking (current/past/never)%	36.4% past (45 ± 4 pack/year)^∗^/18.2% never
FEV1 (mean ± standard deviation)	81.6% ± 16.6
FVC (mean ± standard deviation)	89.1% ± 11.1
DLCO (mean ± standard deviation)	73.1% ± 10.5
Cardiac comorbidities (50% of pts)	Hypertension (50% of pts) Arrhythmia (20% of pts)
Other comorbidities (80% of pts)	COPD (63.6%) Dyslipidemia (36.4%) Diabetes (27.3%) Previous malignancy (27.3%) Cerebrovascular disease (18.2%) Obesity with OSAS (9%) Previous tuberculosis (9%) Peripheral vascular disease (9%) HCV-related liver disease (9%) Psoriasis (9%)
Extent of lung resection^∗∗^	Lobectomy (*n* = 8)^∗∗∗^ Bilobectomy (*n* = 1) Pneumonectomy (*n* = 1) Segmentectomy (*n* = 1)^∗∗∗∗^
Histology (%)	Adenocarcinoma (63.6%) Squamous cell carcinoma (36.4%)
p-stage^∗∗∗∗∗^	IA (36.4%) IB (18.2%) IIA (9%) IIIA (36.4%)

^∗^Past smokers quit at least 1 year before. ^∗∗^The lung resection was always associated with systematic mediastinal lymphadenectomy. After surgery, four patients received adjuvant chemotherapy and two patients received radiation therapy. ^∗∗∗^One patient submitted to lobectomy had been submitted to a previous, homolateral, lobectomy. ^∗∗∗∗^The patient submitted to (lingular) segmentectomy had been previously submitted to bilobectomy. ^∗∗∗∗∗^Pathological staging was established according to the Eighth Edition of the IASLC TNM Staging System for Lung Cancer.

**(a):** 

Patients non-R	0 months (T0)	6 months (T1)	12 months (T2)	18 months (T3)	24 months (T4)
1	33.88	33.84	33.23	33.7	33.81
2	33.81	33.27	32.58	32.39	32.48
3	32.51	31.93	31.98	32.01	32.19
4	32.47	32.5	32.54	31.88	32.18
5	32.26	32.84	32.88	32.49	32.75
8	32.04	32.01	31.58	31.87	31.82

**(b):** 

PatientsR	0 months (T0)	6 months (T1)	12 months (T2)	18 months (T3)	24 months (T4)
6	33.15	33.19	33.89	34.45	34.78
7	33.06	33.37	32.45	32.66	33.06
9	32.39	32.03	32.03	33.801	34.76
10	32.67	32.69	32.90	33.40	34.90
11	32.44	32.01	31.58	31.87	33.55

**Table 3:** Follow-up results and outcome of the patients enrolled.

Follow-up period		24 months
Outcome	Alive/deceased	9/2
	Recurrence	No 54.6% Yes 45.4%: local 27.3% Distant 18.1%
	Median disease-free interval	20 months

## Data Availability

Source data and material will be made available upon reasonable request.

## Abbreviations

AIOM: Associazione Italiana di Oncologia Medica
CEA: Carcinoembryonic antigen
COPD: Chronic obstructive pulmonary disease
EBT: Exhaled breath temperature
IASLC: The International Association for the Study of Lung Cancer
NSCLC: Non-small-cell lung cancer
ROC: Receiver operating characteristic
SD: Standard deviation
STROBE: Strengthening the Reporting of Observational Studies in Epidemiology
TNM: Tumor-node-metastasis
VEGF: Vascular endothelial growth factor.
